# Effects of Butyric Acid Supplementation on the Gut Microbiome and Growth Performance of Weanling Pigs Fed a Low-Crude Protein, Propionic Acid-Preserved Grain Diet

**DOI:** 10.3390/microorganisms13030689

**Published:** 2025-03-19

**Authors:** Kathryn Ruth Connolly, Torres Sweeney, Marion T. Ryan, Stafford Vigors, John V. O’Doherty

**Affiliations:** 1 School of Agriculture and Food Science, University College Dublin, Belfield, D04 W6F6 Dublin, Ireland; ruth.connolly@ucdconnect.ie (K.R.C.); staffordvigors1@ucd.ie (S.V.); 2 School of Veterinary Medicine, University College Dublin, Belfield, D04 W6F6 Dublin, Ireland; torres.sweeney@ucd.ie (T.S.); marion.ryan@ucd.ie (M.T.R.)

**Keywords:** piglets, weaning, organic acids, butyric acid, SCFAs, cereal preservation

## Abstract

Reducing crude protein (CP) in weaner pig diets lowers post-weaning diarrhoea risk but may impair growth performance. This study aimed to identify the beneficial effects of organic acid (OA)-preserved grain and butyric acid supplementation on gut health and growth in low-CP diets. At harvest, grain was divided into two batches: one dried at 65 °C, the other treated with a propionic acid. Ninety-six piglets (28 days old) were assigned to four treatments: (1) dried grain, (2) OA-preserved grain, (3) dried grain + 3% butyric acid, and (4) OA-preserved grain + 3% butyric acid. On day 8, microbial composition, inflammatory markers, volatile fatty acids, and intestinal morphology were assessed. The OA-preserved grain improved feed conversion ratio (*p* < 0.05) increased beneficial gut bacteria (*p* < 0.01), elevated caecal butyrate (*p* < 0.05), reduced jejunal *CXCL8* expression (*p* < 0.05), and enhanced nutrient digestibility (*p* < 0.01). Butyric acid reduced feed intake (*p* < 0.05), improved nutrient digestibility (*p* < 0.01), decreased colonic *Proteobacteria* (*p* < 0.05), and increased colonic propionate and butyrate (*p* < 0.01). Combining OA-preserved grain with butyric acid elevated ileal *Proteobacteria* and *Pasteurellaceae* (*p* < 0.05). In conclusion, while OA-preserved grain improves feed efficiency, nutrient digestibility, and gut microbiota, supplementing butyric acid enhances nutrient digestibility but reduces feed intake, and their combination may disrupt the microbial balance.

## 1. Introduction

Weaned pigs are often offered high crude protein (CP) diets (20–23%) to compensate for low feed intake and to promote growth [1,2]. However, insufficient digestive enzyme production often leads to undigested protein reaching the colon, increasing pathogenic microbial proliferation [3], proteolytic fermentation [4] and intestinal pH [5]. This fermentation produces toxic metabolites, reducing gastrointestinal structure and function and contributes to post-weaning diarrhoea (PWD) [3,6,7,8], which has traditionally been managed by adding zinc oxide to the diet. Following the 2022 EU ban on the prophylactic use of in-feed medications (Commission Implementing Decision of 26 June 2017, C (2017) 4529 Final; Regulation (EU) June 2019), the reduction of dietary CP is being extensively researched in the post-weaning period. While low-CP diets can reduce PWD [9,10,11], they can also slow growth and hinder intestinal development [12], possibly due to reduced nutrient digestibility and also reduced proliferation and fermentation of intestinal short-chain fatty acid (SCFAs)-producing bacteria [13]. Hence the need to optimise low-CP diets further in relation to the growth performance and intestinal health of post-weaned pigs.

One strategy to improve nutrient digestibility in low-CP diets is the incorporation of organic acids (OA). Supplementing OAs can lower gastrointestinal pH, increase enzyme activity and improve protein digestion, while also promoting the proliferation of beneficial gut bacteria such as *Lactobacillus* while reducing pathogens such as *Escherichia coli* [14]. Additionally, OAs, particularly propionic acid [15], are effective grain preservatives, reducing mycotoxin and mould contamination [16]. Recent studies have reported that OA-preserved grain increases the ileal digestibility of nitrogen and improves the growth performance of post-weaned pigs [17]. Therefore, OA-preserved low-CP diets could improve protein digestion, intestinal health and growth performance in post-weaned pigs, potentially reducing the limitations associated with low-CP diets [18].

An alternative strategy to optimise low-CP diets in post-weaned pigs is the supplementation of exogenous butyrate, which can help compensate for the reduced intestinal butyrate production associated with these diets. Butyrate is produced by microbial fermentation [19] and is the preferred energy source of colonocytes [20]. Beyond its metabolic function, butyrate has anti-inflammatory, anti-oxidant and anti-diarrheal properties, supporting the intestinal mucosal barrier [21], architecture [22] and the proliferation of beneficial gut bacteria [23]. Furthermore, butyrate supplementation has been shown to slow gastric emptying in weaned pigs, potentially improving digestive efficiency [20]. Supplementing low-CP diets with butyrate may increase intestinal butyrate concentrations and improve nutrient digestion, gut function, and microbiome balance, thereby enhancing growth performance and intestinal health in post-weaned pigs. This strategy could help address the limitations of low-CP diets, such as reduced nutrient digestibility and slower intestinal development, supporting both gut health and growth in the absence of high-CP levels.

Given these potential benefits, combining OA-preserved grain with butyric acid supplementation in low-CP diets could improve protein digestibility and increase intestinal SCFA concentrations, mitigating the adverse effects on growth performance and intestinal health in weaned pigs. Therefore, the objective of this study was to examine the effects of OA-preserved grain and butyric acid supplementation in low-CP diets on the growth performance and intestinal microbiome of post-weaned pigs. The hypothesis of this study was firstly that the individual use of OA-preserved grain or butyric acid supplementation would improve the growth performance and intestinal microbiome of post-weaned pigs offered low-CP diets and that secondly, the combination of OA-preserved grain and butyric acid supplementation in low-CP diets would have the greatest effect on piglet performance and health, due to their synergetic effects.

## 2. Materials and Methods

All the experimental procedures described in this study were approved under the University College Dublin Animal Research Ethics Committee (AREC-22-02-O’Doherty) and were conducted in accordance with Irish legislation (*SI* no 543/2012) and the EU directive 2010/63/EU for animal experimentation.

### 2.1. Grain Management and Quality Assessment

Winter wheat (cv. *JB Diego*) and spring barley (cv. *SY Errigal*) grains obtained from McAuley Feeds (Burtontown, Co., Meath, Ireland) were used in this study and underwent the same management and preservation procedures described previously by Maher et al. [17]. The grains were grown and harvested in the 2021 season. The winter wheat was sown in October 2020, adhering to recommended practices such as a three-spray fungicide program and a three-part nitrogen (N) application at a rate of 180 kg/N/ha. The wheat was harvested in August 2021 during optimal weather conditions, which resulted in a moisture content of 180 g/kg. The spring barley was planted in March 2021 and followed the recommended practices such as a two-spray fungicide program and a two-split N application rate of 140 kg/ha. The spring barley was harvested with a moisture content of 181 g/kg in August 2021. Prior to storage, both grain types were separated into two batches. One batch was dried with a continuous flow-type dryer (Cimbria, Thisted, Denmark) at 65 °C for 3 h before being allowed to cool for 2 h. The moisture content of the winter wheat and spring barley following the drying process was 140.0 g/kg and 140.5 g/kg, respectively. The second batch of grain was preserved using a propionic acid mould inhibitor, specifically a liquid surfactant (MycoCURB© ES Liquid; propionic acid (650 g/kg), ammonium propionate (70 g/kg), glycerol polyethyleneglycol ricinoleate (17.5 g/kg) and a carrier). The propionic acid mould inhibitor was acquired from Adesco Nutricines, (Dungarvan, Co., Waterford, Ireland) and applied using spray action at an inclusion level of 4 g/kg. To ensure even distribution of the acid, a mixing auger was used. Following preservation, all batches of grain were ventilated and stored prior to diet manufacture.

Grain quality was determined at the time of harvest. Moisture content was assessed using a DICKEY-john GAC 2500_UGMA electronic moisture meter (DICKEY-john, Auburn, IL, USA). At the time of diet formulation, the grab sample technique was used to collect representative samples of all dietary treatments. These samples were subsequently analysed for gross energy (GE), dry matter (DM), ash, crude fibre, CP and mycotoxins. The concentrations of the mycotoxins aflatoxin B1, B2, G1 and G2, fumonisin B1 and B2, deoxynivalenol (DON), T-2 toxin, HT-2 toxin, zearalenone (ZEN) and ochratoxin A (OTA) were determined using liquid chromatography–mass spectrometry as previously described by Soleimany et al. [24]. The chemical and mycotoxin analyses of the wheat and barley post-storage are presented in Table 1.

### 2.2. Experimental Design and Dietary Treatments

This study comprised four dietary treatments in a 2 × 2 factorial design. Ninety-six piglets (progeny of Meatline Hermitage boar (Sion Road, Kilkenny, Ireland) × (Large White × Landrace sow)) were selected from a commercial swine unit on the day of weaning (28 days), with an average live weight of 7.4 kg ± 0.81 kg (standard deviation). The piglets were subsequently blocked on the basis of live weight, litter of origin and sex, and within each block, were allocated to one of four dietary treatments for the entire 35-day experimental period. Piglets were offered stage 1 diets for the initial 15 days of the experiment: (1) dried diet, (2) OA-preserved diet, (3) dried diet with 3% encapsulated butyric acid and (4) OA-preserved diet with 3% encapsulated butyric acid. The stage 1 dried diet and OA-preserved diet consisted of 458 g/kg of grain with 308 g/kg being either dried or OA-preserved wheat and 150 g/kg being dried or OA-preserved barley. The stage 1 dried diet with 3% encapsulated butyric acid and OA-preserved diet with 3% encapsulated butyric acid consisted of 455 g/kg of grain, with 305 g/kg being either dried or OA preserved wheat and 150 g/kg being dried or OA preserved barley. The encapsulated butyric acid ButiPEARL^®^ (Kemin Industries, Des Moines, IA, USA), was included at a rate of 3 g/kg. The remaining 542 g/kg of the composition comprised a concentrate obtained from Cargill (Naas, Co., Kildare, Ireland) as outlined in Table 1. After, 15 days the piglets were offered a corresponding stage 2 diet for the remainder of the study (d 15–35): (1) dried diet, (2) OA-preserved diet, (3) dried diet with 3% encapsulated butyric acid and (4) OA-preserved diet with 3% encapsulated butyric acid. The stage 2 dried diet and OA-preserved diet were comprised of 536 g/kg of grain with 386 g/kg being either dried or OA-preserved wheat and 150 g/kg being dried or OA-preserved barley. The stage 2 dried diet with 3% encapsulated butyric acid and OA-preserved diet with 3% encapsulated butyric acid were comprised of 533 g/kg of grain with 383 g/kg being either dried or OA-preserved wheat and 150 g/kg being dried or OA preserved barley. The encapsulated butyric acid was included at 3 g/kg. The remainder of the composition (464 g/kg) consisted of a concentrate sourced from Cargill (Naas, Co Kildare, Ireland) as outlined in Table 2. Celite (5 g/kg) was added to the stage 2 diets at diet manufacture for the measurement of the coefficient of apparent total tract digestibility (CATTD) using the acid-insoluble ash (AIA) method, detailed by McCarthy et al. [25]. The stage 1 diets were formulated to contain similar levels of standard ileal digestible lysine (13.0 g/kg), net energy (11.0 MJ/kg) and CP (190 g/kg). The stage 2 diets were also formulated to contain similar levels of standard ileal digestible lysine (12.0 g/kg), net energy (10.8 MJ/kg) and CP (175 g/kg). Dietary amino acid (AA) levels were formulated to meet or exceed requirements of the NRC [1]. The concentrate contained synthetic lysine, methionine, threonine, tryptophan and valine to meet AA requirements [1]. The diets were milled on-farm and offered as meal. The ingredient and chemical analysis of the dietary treatments are outlined in Table 2 and Table 3.

### 2.3. Animal Management

The piglets were housed in fully slated pens (1.68 × 1.22 m) in groups of three. The temperature of the houses was thermostatically set at 30 °C for the first week and then reduced by 2 °C per week. The humidity was maintained at 65%. Feed and water were available ad libitum from two-space feeders and nipple drinkers. The piglets were initially weighed at the start of the experiment (day 0) and then every seven days to calculate average daily gain (ADG). Offered feed was also weighted every seven days to calculate average daily feed intake (ADFI) and feed conversion ratio (FCR).

### 2.4. Faecal Scoring

Faecal scoring was carried out every day in the morning and evening by the same operator using a scale system ranging from 1 to 5: 1 = hard, firm faeces; 2 = slightly soft faeces; 3 = soft, partially formed faeces; 4 = loose, semi-liquid faeces; and 5 = watery, mucous-like faeces, as described previously by Connolly et al. [26].

### 2.5. Sample Collection

On the 8th day post-weaning, one piglet from every pen (*n* = 8) was humanely euthanised for the purpose of sample collection. The piglets received a lethal injection of pentobarbitone sodium (Euthanal solution, 200 mg/mL; Chanelle Pharma, Galway, Ireland) at a level of 0.7 mL/kg of body weight into the cranial vena cava. The euthanasia was performed by a competent individual, out of the sight and sound of the other piglets. Immediately after death, the entire gastrointestinal tract was carefully removed.

Sections from the different intestinal sites: the duodenum (10 cm from the stomach), the jejunum (60 cm from the stomach) and the ileum (15 cm from the caecum) were excised and preserved in Formalin 10%, Q Path^®^ buffered, (VWR, Leicestershire, UK). Tissue samples (1 cm) were dissected from the duodenum, jejunum and ileum for RNA isolation, to determine the relative expression of genes related to nutrient transportation, inflammatory cytokines, pathogen recognition receptors and mucins using QPCR. The tissue samples were dissected along the mesentery before being emptied and rinsed using sterile phosphate-buffered saline (Oxoid, Hampshire, UK). The samples were then stripped of the overlying smooth muscle prior to storage in 5 mL of RNAlater^®^ solution (Applied Biosystems, Foster City, CA, USA) overnight at 4 °C. The RNAlater^®^ was then removed before the samples were stored at −80 °C. Ileal and colonic digesta were collected and stored in sterile containers (Sarstedt, Wexford, Ireland) on dry ice before being stored at −80 °C for 16s rRNA analysis. Similarly, digesta from the caecum and colon was collected and stored in sterile containers (Sarstedt, Wexford, Ireland) on dry ice, before storage at −20 °C for volatile fatty acid (VFA) analysis. On the 30th day post-weaning, faeces samples were collected to determine the CATTD of nutrients. The internal marker AIA was used to calculate the CATTD [25]. The following equation was used to calculate the CATTD of nutrients: (1 − [nutrients in faeces/nutrient in diet] × [AIA-diet/AIA-faeces]). The nutrient concentrations in faeces and diet refer to the nutrient content (g/kg) in the DM, respectively. The AIA-diet and AIA-faeces represent the concentrations of AIA in the DM of the diet and faeces [27].

### 2.6. Feed and Faecal Analysis

At the time of diet formulation, representative samples from the stage 1 and stage 2 diets of each dietary treatment were collected. Faecal samples were collected from every pen on day 30 post-weaning and immediately frozen at −20 °C. The feed samples were milled through a 1 mm screen (Christy and Norris Hammer Mill, Chelmsford, UK). The DM of the feed and faeces samples was then determined by drying them for 72 h at 55 °C. Weighted samples were then ignited for 6 h at 550 °C in a muffle furnace (Nabertherm, New Castle, DE, USA) to determine dietary crude ash content. The GE of the feed and faeces was assessed with an adiabatic bomb calorimeter (Parr Instruments, St Moline, IL, USA). Dietary nitrogen content was assessed utilising the Leco FP 528 instrument (Leco Instruments Ltd., Stockport, UK), while the dietary AA concentrations were determined using an HPLC as detailed by Iwaki et al. [28]. The crude fat levels of the diets were assessed using light petroleum ether and Soxtec instrumentation (Tecator, Hillerod, Sweden). Dietary crude fibre levels were assessed in accordance with the AOAC 1990 methodology (number 978.10)) [29]. The neutral detergent fibre (NDF) content of the feed was assessed utilising the Ankom 220 Fibre Analyser (Ankom Technology, Macedon, NY, USA) as detailed by Van Soest et al. [30]. The chemical analysis of the dietary treatments is presented in Table 3.

### 2.7. Gut Morphological Analysis

Tissue samples from the duodenum, jejunum and ileum were prepared for gut morphological assessment with the use of standard paraffin embedding techniques as outlined previously by Rattigan et al. [31]. Each sample was sectioned to a thickness of 5 μm and stained with haematoxylin–eosin. Villus height (VH) and crypt depth (CD) were measured using a light microscope equipped with an image analyser (Image-Pro Plus; Media Cybernetics, Oxon, UK). A minimum of fifteen measurements of intact and well-orientated villi and crypts were taken per tissue per animal.

### 2.8. Gene Expression in the Small Intestine

#### 2.8.1. RNA Extraction and cDNA Synthesis

Total RNA was extracted from tissue collected from the duodenum, jejunum and ileum using TRI Reagent (Sigma-Aldrich, St. Louis, MO, USA), before being further purified using the GenElute™ Mammalian Total RNA Miniprep kit (Sigma-Aldrich, St. Louis, MO, USA). A DNase step was incorporated using an on-column DNase 1 Digestion set (Thermo Scientific, Waltham, MA, USA) as described previously by Kiernan et al. [32]. Total RNA (2 μg) was reverse transcribed using a High-capacity cDNA Reverse Transcription Kit (Applied Biosystems, Foster City, CA, USA) and random primers in a final reaction volume of 40 μL in accordance with the manufacturer’s guidelines. Nuclease-free water was then used to make up the cDNA volume to 360 μL.

#### 2.8.2. Quantitative Real-Time Polymerase Chain Reaction (QPCR)

The QPCR reaction mixture (20 μL), consisted of 10 μL of GoTaq Master Mix (Promega, Madison, WI, USA), 1.2 μL of forward and reverse primers (5 μM), 3.8 μL of nuclease-free water and 5 μL of cDNA. The QPCR reactions were all carried out in duplicates using the 7500 ABI Prism Sequence Detection System (Applied Biosystems, Foster City, CA, USA). The thermal cycling conditions included an initial denaturation step for 10 min at 95 °C, followed by 40 cycles at 95 °C (15 s) and 60 °C (60 s). The primers were designed using Primer Express Software v3.01 (Applied Biosystems, Foster City, CA, USA) and subsequently manufactured by Eurofins (Milton Keynes, UK). The specificity was verified from the dissociation curves. The QPCR assay efficiencies were determined by plotting the cycling threshold values which resulting from four-fold serial dilutions of cDNA against their arbitrary quantities. Only assays which demonstrated single products and 90–100% efficiency were accepted. Normalised relative quantities were obtained using qbase PLUS software v2.0 (Biogazelle, Ghent, Belgium) from stable reference genes: *HMBS*, *H3F3A* and *YWHAZ*. These genes were selected as gastrointestinal reference genes based on their M value (<1.5) generated by the GeNorm algorithm within GeNorm.

The target genes analysed in the small intestine are presented in Table 4. These include *FABP2*, *SLC2A1*, *SLC15A1*, *IL1A*, *IL1B*, *IL6*, *CXCL8*, *IL17*, *IL22*, *TNF*, *FOXP3*. *CLDN1*, *TJP1 MUC2*, and *TLR4*.

### 2.9. Volatile Fatty Acid Analysis

Concentrations of VFAs in the caecal and colonic digesta were determined with the use of gas chromatography as described previously by Clarke et al. [27]. One gram of digesta was diluted with 2.5× the sample weight and centrifuged using a Sorvall GLC-2B centrifuge (DuPont, Wilmington, DE, USA) at 1400× *g* for 10 min. After centrifugation, 1 mL of internal standard (0.05% 3-methyl-n-valeric acid in 0.15 M oxalic acid dihydrate) was mixed with 3 mL of distilled water and 1 mL of supernatant and then centrifuged at 500× *g* for 10 min. The resulting supernatant was filtered through a syringe filter (0.45 polytetrafluorethylene (TFE)) into a chromatographic sample vial. One μL of this mixture was injected into a Varian 3800GC (Markham, ON, Canada) with an ECTM 1000 Grace column (15 m × 0.53 mm I.D) with a film thickness of 1.20 μm. The temperature program was configured as follows: it started at 95 °C at a rate of 3 °C per minute. Then, the temperature increased from 95 °C to 200 °C at a rate of 20 °C per minute and was maintained at 200 °C for 0.50 min. The detector temperature was set to 280 °C, and the injector temperature was set to 240 °C.

### 2.10. Microbiological Analysis

#### 2.10.1. Microbial DNA Extraction

The microbial genomic DNA was extracted from the caecal and colonic digesta using the QIAamp Powerfecal Pro DNA kit (Qiagen, West Sussex, UK) according to the manufacturer’s guidelines. A Nanodrop ND-1000 Spectrophotometer (Thermo Scientific, Wilmington, DE, USA) was used to measure to quality and quantity of the isolated DNA.

#### 2.10.2. Illumina Sequencing

An Illumina MiSeq platform was used in accordance with protocol (Eurofins Genomic, Eberberg, Germany) to perform high throughput sequencing of the V3–V5 hypervariable region of the bacterial 16s rRNA gene. Universal primers containing adapter overhang nucleotide sequences for forward and reverse index primers were used for the PCR amplification of the V3–V5 region. AMPure XP beads (Beckman Coulter, Indianapolis, IN, USA) were utilised to purify the amplicons which were then prepared for Index PCR using Nextera XT index primers (Illumna, San Diego, CA, USA). The indexed samples were then purified with AMPure XP beads before quantification with a fragment analyser (Agilent, Santa Clara, CA, USA). Equal quantities from the samples were then pooled and quantified with the Bioanalyser 7500 DNA kit (Agilent, Santa Clara, CA, USA) and sequenced using the v3 chemistry (2 × 300 bp paired-end reads).

#### 2.10.3. Bioinformatics

Eurofins Genomics (Eberberg, Germany) performed the bioinformatic analysis of the sequences with the use of the open-source package Quantitative Insights into Microbial Ecology (Version 1.9.1) [33]. Raw reads which passed the standard Illumina chastity filter were demultiplexed in accordance with their index sequences (read quality > 30). The primer sequences were trimmed from the beginning of the raw forward and reverse reads. Reads were discarded if the primer sequences did not match perfectly, ensuring that only high-quality reads were retained. The software Flash 2.200 was used to merge paired-end reads to generate a single, longer read that covers the entire target region [34]. To avoid false-positive merges, the pairs were merged with a minimum overlap size of 10 bp. If merging was impossible, then forward reads were maintained for the subsequent analysis. The merged reads were then quality filtered according to the expected and known length variation in the V3–V5 region. Retained forward reads were cut at the end to a total length of 300 bp to remove any low-quality bases. Both merged and retained reads comprised of ambiguous reads were discarded. The filtered reads were then used to generate the microbiome profile. Chimeric reads were identified and discarded using the de-novo algorithm of UCHIIME [35], as implemented in the VSEARCH package [36]. The remaining set of high-quality reads was processed with minimum entropy decomposition (MED) to sort the reads into operational taxonomic units (OTU) [37]. Taxonomic assignment of each OTU was performed by aligning cluster representative sequences to the NCBI nucleotide sequence database using DC-MEGABLAST. A sequence identity of 70% across at least 80% of the representative sequence was required to be considered as a reference sequence. Normalisation of the bacterial taxonomic units was achieved using linear-specific copy numbers of the relevant marker genes to improve estimates [38]. The data matrix, consisting of the normalised OTU table combined with the phenotype metadata and phylogenetic tree, was loaded into the phyloseq package in R (Version 3.5.0). Differential abundance testing was carried out on the tables from phyloseq at phylum, family and genus levels.

The dynamics of richness and diversity in the microbiome were calculated using the observed, Fisher, Shannon and Simpson indices. These diversity indices assign different weights to the parameters of richness and evenness. Richness represents the number of distinct taxa observed in a sample but does not account for their frequency of occurrence. While evenness compared the similarity of the population size of each of the species present in a sample [39]. The observed alpha diversity measures species richness, whereas the Fisher, Shannon and Simpson indices account for both richness and evenness [39,40]. Beta diversity measurements measure the separation of the OTU phylogenetic structure in a sample compared to all other samples. This was achieved by normalising the data to allow for comparison of taxonomic feature counts across all samples. The non-phylogenetic distance metrics Bray Curtis was utilised in phyloseq in R [38,41].

### 2.11. Statistics

The growth performance parameters (ADFI, ADG, FCR, and body weight (BW)) were analysed using the PROC GLM procedure of SAS^®^ software version 9.4 (SAS Institute, Inc., Cary, NC, USA), (not Bonferroni adjusted). The analysis was performed for periods: d 0–21, d 22–35 and for the overall experimental period. The statistical model incorporated grain preservation method, butyric acid supplementation, and their associated two interactions. Initial body weight was used as a covariate. The faecal scores were analysed using repeated measures analysis using the PROC MIXED procedure. The statistical model incorporated grain preservation method, butyric acid supplementation, time, and their associated two- and three-way interactions. The experimental unit for performance and FS data was the pen. The PROC GLM procedure was used to analyse the intestinal VFA, gene expression data (Bonferroni adjusted *p* < 0.05) and bacterial alpha diversity data. The PROC GLIMMIX procedure for nonparametric data was used to analyse the microbiome, with the results presented as least square means, using Benjamini–Hochberg (BH) adjusted *p*-values. The results are displayed as least square means along with their standard errors. The probability level that denotes significance is *p* < 0.05 while a numerical tendency is between *p* > 0.05 and *p* < 0.10.

## 3. Results

### 3.1. Grain Quality

The chemical and microbial analysis of the dried and OA-preserved wheat and barley at the time of diet formulation are presented in Table 1. The OA-preserved wheat and barley both had lower DM contents in comparison to the dried wheat and barley. The dried barley had increased levels of T-2 toxin, HT-2 toxin and OTA compared to OA-preserved barley. The dried wheat had increased levels of OTA compared to OA-preserved wheat.

### 3.2. Growth Performance and Faecal Scores

The effects of grain preservation method and butyric acid supplementation on BW, ADG, ADFI, FCR and FS during the post-weaning period (d 0–15, d 15–35 and d 0–35) are presented in Table 5.

During the first 15 days, the supplementation of butyric acid reduced ADFI compared to non-butyric acid-supplemented diets (416 vs. 450 g/day, SEM 11.06; *p* < 0.05). There was no effect of grain preservation method or butyric acid supplementation on ADG, FCR or BW during days 0–15. During days 15–35, there was an interaction between the grain preservation method and butyric acid supplementation on ADFI (*p* < 0.05). The supplementation of butyric acid reduced ADFI in OA-preserved grain but had no effect on ADFI in dried grain. There was no effect of the grain preservation method on ADG, FCR, FS or BW from day 15 to 35. During the overall experimental period (days 0–35), the supplementation of butyric acid reduced ADFI compared to non-butyric acid-supplemented diets (0.656 vs. 0.695, SEM 14.07; *p* < 0.05). Piglets offered the OA-preserved grain had improved FCR compared to those offered dried grain diets (1.39 vs. 1.49, SEM 0.033; *p* < 0.05).

### 3.3. Coefficient of Apparent Total Tract Digestibility

The effect of the grain preservation method and butyric acid supplementation on the CATTD of nutrients on day 30 post-weaning are presented in Table 6. Piglets offered the OA-preserved grain had improved CATTD of DM (0.856 vs. 0.846, SEM 0.0028), OM (87.44 vs. 86.39, SEM 0.262) and GE (85.17 vs. 83.97, SEM 0.289) compared to dried grain (*p* < 0.05). Piglets supplemented with butyric acid had increased CATTD of N (81.10 vs. 79.65, SEM 0.486) and GE (85.02 vs. 84.13, SEM 0.289) compared to non-butyric acid-supplemented piglets. There was an interaction between the grain preservation method and butyric acid supplementation on the CATTD of ash (*p* < 0.05). The supplementation of butyric acid increased the CATTD of ash in OA-preserved grain, but there was no effect on the CATTD of ash when butyric acid was supplemented to the dried grain diets.

### 3.4. Small Intestinal Morphology

The effects of grain preservation method and butyric acid supplementation on intestinal morphology are presented in Table 7. In the duodenum, piglets offered OA-preserved grain had increased VH compared to dried grain (306.13 vs. 258.19 μm, SEM 14.029; *p* < 0.05). The supplementation of butyric acid reduced CD compared to non-butyric supplemented diets (101.76 vs. 129.83 μm, SEM 6.558; *p* < 0.01). The supplementation of butyric acid increased the VH:CD ratio compared to non-butyric supplemented diets (2.28 vs. 2.87, SEM 0.186; *p* < 0.05). In the jejunum, there was an interaction between the grain preservation method and butyric acid supplementation on VH (*p* < 0.05). The OA-preserved grain + butyric acid diet had increased VH compared to the dried grain + butyric acid diet, however, there was no effect of grain preservation on VH in the unsupplemented diets. There was no effect of grain preservation method or butyric acid supplementation on CD or VH:CD ratio in the jejunum. There was no effect of the grain preservation method of butyric acid supplementation on ileal VH, CD or VH:CD ratio.

### 3.5. Gene Expression Analysis

Genes involved in nutrient transportation, mucosal barrier function and immunity in the small intestine which were differentially expressed are presented in Table 8.

In the duodenum, there was an interaction between the grain preservation method and butyric acid supplementation on the relative expression of *MUC2* (*p* = 0.05). The supplementation of butyric acid increased the relative expression of *MUC2* in the OA-preserved grain diet, however, there was no effect of butyric acid supplementation on *MUC2* in the dried grain diet. There was no effect of grain preservation method or butyric acid supplementation on the relative expression of genes related to nutrient transportation, mucosal barrier function or immunity in the duodenum (*p* > 0.05).

In the jejunum, there was an interaction between the grain preservation method and butyric acid supplementation on the relative expression of *SLC2A1* (*p* < 0.05). The supplementation of butyric acid increased the relative expression of *SLC2A1* in the OA-preserved grain diet, but there was no effect of butyric acid supplementation on *SLC2A1* in the dried grain diet. Piglets offered OA-preserved grain had reduced relative expression of *IL17* (2.22 vs. 0.97, SEM 0.390) and *CXCL8* (0.89 vs. 1.26, SEM 0.123) compared to dried grain (*p* < 0.05). Piglets offered OA-preserved grain had increased relative expression of *TJP1* compared to dried grain (1.03 vs. 0.94, SEM 0.029; *p* < 0.05). There was no effect of butyric acid supplementation on the expression of genes related to nutrient transportation, mucosal barrier function or immunity in the jejunum (*p* > 0.05).

In the ileum, there was an interaction between the grain preservation method and butyric acid supplementation on the relative expression of *TLR4* (*p* < 0.01). The supplementation of butyric acid reduced the relative expression of *TLR4* in the dried grain diet, but there was no effect of butyric acid supplementation on *TLR4* in the OA-preserved grain diet. There was no effect of grain preservation method or butyric acid supplementation on the relative expression of genes related to nutrient transportation, mucosal barrier function or immunity in the ileum (*p* > 0.05).

### 3.6. Differential Bacterial Abundance Analysis

#### 3.6.1. Bacterial Richness and Diversity

There was no effect of the grain preservation method or butyric acid supplementation on the Observed, Fisher, Shannon or Simpson index measures of diversity in the ileal and colonic digesta (*p* > 0.05). There were no differences in Beta diversity in both the ileal and colonic digesta microbiome based on visualisation using the Bray Curtis distance matrix and multi-dimensional scaling. This indicates variation between the individual piglets within the dietary treatments in this experiment.

#### 3.6.2. Differently Abundant Phlya

The effects of grain preservation method and butyric acid supplementation on the relative abundance of bacterial phyla are presented in Table 9.

In the ileum, the predominant phyla in all treatments were Firmicutes (~88.28%) and Proteobacteria (~8.59%).

There were interactions between the grain preservation method and butyric acid supplementation on the relative abundance of Firmicutes and Proteobacteria (*p* < 0.01). The supplementation of butyric acid reduced the relative abundance of Firmicutes in the dried grain diet, however, there was no effect of butyric acid supplementation on Firmicutes in the OA-preserved grain diet. The supplementation of butyric acid reduced the relative abundance of Proteobacteria in the dried grain diet, but the supplementation of butyric acid increased Proteobacteria in the OA-preserved grain diet.

In the colon, the predominant phyla were Firmicutes (~74.97%), Bacteroidetes (~12.06%), Actinobacteria (~4.19%), Spirochaetes (~2.19%), Tenericutes (~1.25%) and Proteobacteria (~1.23%).

There was an interaction between the grain preservation method and butyric acid supplementation on the relative abundance of Bacteroidetes (*p* < 0.05). The OA-preserved grain diet had an increased relative abundance of Bacteroidetes compared to the dried grain diet, however, there was no effect of grain preservation on Bacteroidetes in the butyric acid-supplemented diets. Piglets offered OA-preserved grain had an increased relative abundance of Tenericutes compared to dried grain (2.02 vs. 0.48, SEM 0.356; *p* < 0.01). Piglets offered OA-preserved grain had reduced relative abundance of Actinobacteria (2.99 vs. 5.39, SEM 0.580) and Spirochaetes (0.93 vs. 3.46, SEM 0.465) compared to dried grain (*p* < 0.05). The supplementation of butyric acid increased the relative abundance of Spirochaetes compared to non-butyric supplemented diets (3.35 vs. 1.04, SEM 0.457; *p* < 0.05). The supplementation of butyric acid decreased the relative abundance of Proteobacteria compared to non-butyric supplemented diets (0.29 vs. 2.16, SEM 0.368; *p* < 0.01).

#### 3.6.3. Differently Abundant Families

The effects of grain preservation method and butyric acid supplementation on the relative abundance of bacterial families are presented in Table 10.

In the ileum, there were interactions between the grain preservation method and butyric acid supplementation on the relative abundance of *Clostridiaceae* and *Streptococcaceae* (*p* < 0.05). The supplementation of butyric acid increased the relative abundance of *Clostridiaceae* in the dried grain diet but reduced *Clostridiaceae* in the OA-preserved grain diet. The supplementation of butyric acid increased the relative abundance of *Streptococcaceae* in the dried grain diet, however, butyric acid supplementation had no effect on *Streptococcaceae* in the OA-preserved grain diet. Piglets offered OA-preserved grain had increased relative abundance of *Pasteurellaceae* compared to dried grain (5.24 vs. 0.42, SEM 0.724; *p* < 0.05). The supplementation of butyric acid increased the relative abundance of *Pasteurellaceae* compared to non-butyric supplemented diets (4.88 vs. 0.78, SEM 0.641; *p* < 0.05).

In the colon, there were interactions between the grain preservation method and butyric acid supplementation on the relative abundance of *Rikenellaceae* and *Prevotellaceae* (*p* < 0.05). The OA-preserved grain diet had increased relative abundance of *Rikenellaceae* and *Prevotellaceae* compared to the dried grain diet, but there was no effect of the grain preservation method on *Rikenellaceae* and *Prevotellaceae* in the butyric acid-supplemented diets. Piglets offered OA-preserved grain had increased relative abundance of *Spiroplasmataceae* (1.10 vs. 0.324 SEM 0.270) and *Hungateiclostridiaceae* (2.82 vs. 1.74, SEM 0.419) compared to dried grain (*p* < 0.05). Piglets offered OA-preserved grain had a reduced relative abundance of *Propionibacteriaceae* (4.38 vs. 5.34, SEM 0.578) and *Spirochaetaceae* (0.95 vs. 2.76, SEM 0.424) compared to dried grain (*p* < 0.05). The supplementation of butyric acid increased the relative abundance of *Spirochaetaceae* compared to non-butyric supplemented diets (2.63 vs. 1.08, SEM 0.413; *p* < 0.05).

#### 3.6.4. Differently Abundant Genera

The effects of grain preservation method and butyric acid supplementation on the relative abundance of bacterial genera are presented in Table 11.

In the ileum, there were interactions between grain preservation method and butyric acid supplementation on the relative abundance of *Lactobacillus*, *Clostridium* and *Streptococcus* in the ileum (*p* < 0.05). The supplementation of butyric acid increased the relative abundance of *Lactobacillus*, *Clostridium* and *Streptococcus* in the dried grain diet, but there was no effect of butyric acid supplementation on *Lactobacillus*, *Clostridium* and *Streptococcus* in the OA-preserved grain diet.

In the colon, there were interactions between the grain preservation method and butyric acid supplementation on the relative abundance of *Gemmiger*, *Anaerocella*, *Alloprevotella* and *Pseudoflavonifractor* (*p* < 0.05). The supplementation of butyric acid increased the relative abundance of *Gemmiger* in the OA-preserved grain diet, however, there was no effect of butyric acid supplementation on *Gemmiger* in the dried grain diet. The OA-preserved grain diet had an increased relative abundance of *Anaerocella* and *Alloprevotella* compared to the dried grain diet, but there was no effect of the grain preservation method on *Anaerocella* and *Alloprevotella* in the butyric acid-supplemented diets. The supplementation of butyric acid reduced the relative abundance of *Pseudoflavonifractor* in the dried grain diet, but there was no effect of butyric acid supplementation on *Pseudoflanonifractor* in the OA-preserved grain diet. Piglets offered OA-preserved grain had increased relative abundance of *Spiroplasma* (1.11 vs. 0.35, SEM 0.271), *Roseburia* (4.00 vs. 2.34, SEM 0.450), *Agathobacter* (1.45 vs. 0.68, SEM 0.301) compared to dried grain (*p* < 0.05). Piglets offered OA-preserved grain had reduced relative abundance of *Propionibacterium* (2.52 vs. 4.98, SEM 0.580), *Dorea* (1.28 vs. 2.81 SEM 0.438), *Blautia* (0.67 vs. 2.41, SEM 0.388), *Ruminococcus* (0.93 vs. 2.19, SEM 0.382) and *Treponema* (0.73 vs. 2.60, SEM 0.411) compared to dried grain (*p* < 0.05). The supplementation of butyric acid reduced the relative abundance of *Propionibacterium* (3.03 vs. 4.47, SEM 0.529) and *Blautia* (0.97 vs. 2.11, SEM 0.363) compared to non-butyric acid-supplemented diets (*p* < 0.05). The supplementation of butyric acid increased the relative abundance of *Treponema* compared to non-butyric acid-supplemented diets (2.36 vs. 0.97, SEM 0.340; *p* < 0.05).

### 3.7. Volatile Fatty Acid 

The effect of grain preservation method and butyric acid supplementation on the molar proportions and total VFA concentrations in the caecal and colonic digesta are presented in Table 12.

In the caecum, there were interactions between the grain preservation method and butyric acid supplementation on the molar proportions of isobutyrate and isovalerate (*p* < 0.05). The OA-preserved grain diet had increased molar isobutyrate and isovalerate proportions compared to dried grain but there was no effect of the grain preservation method on molar isobutyrate and isovalerate proportions in the butyric acid-supplemented diets. Piglets offered OA-preserved grain had increased molar butyrate proportions (0.201 vs. 0.172, SEM 0.0086, SEM; *p* < 0.05) and total VFA concentrations (167.41 vs. 141.55, SEM 7.0209; *p* < 0.05) compared to dried grain. The supplementation of butyric acid reduced molar acetate proportions (0.429 vs. 0.478, SEM 0.0120; *p* < 0.01) and molar propionate proportions (0.272 vs. 0.325, SEM 0.0094; *p* < 0.001) compared to non-butyric supplemented diets.

In the colon, there were interactions between the grain preservation method and butyric acid supplementation on molar proportions of isobutyrate and branched chain fatty acids (BCFAs) (*p* < 0.05) and total. The OA-preserved grain + butyric diet had increased molar isobutyrate and BCFA proportions compared to the dried grain + butyric diet, however, there was no effect of the grain preservation method on molar isobutyrate and BCFA proportions in the unsupplemented diets. The supplementation of butyric acid reduced molar acetate proportions (0.428 vs. 0.505, SEM 0.0089; *p* < 0.001), increased molar propionate proportions (0.330 vs. 0.290, SEM 0.0076; *p* < 0.001) and molar butyrate proportions (0.188 vs. 0.146, SEM 0.0069; *p* < 0.001) compared to non-butyric supplemented diets.

## 4. Discussion

Reducing dietary CP levels can lower the risk of PWD [42,43], but can also compromise growth performance [44,45,46]. To address this challenge, the current study sought to enhance the digestive and fermentative capacity of the intestine by incorporating OA-preserved grain and/or butyric acid into low-CP diets. Offering piglets OA-preserved grain improved FCR during the overall experimental period, increased the CATTD of DM, OM, N and GE, reduced jejunal *CXCL8* expression and increased caecal butyrate proportions compared to dried grain. Butyric acid supplementation reduced ADFI during the overall experimental period, enhanced the CATTD of N and GE, improved duodenal morphology and increased colonic butyrate proportions compared to diets without butyric acid supplementation. Interestingly, the combination diet of OA-preserved grain with butyric acid had the highest abundance of ileal Proteobacteria, *Pasteurellaceae*, and colonic BCFAs on day 8 post-weaning, despite having had the highest CATTD of DM, GE, N, ash and OM of all treatments. These results suggest that OA-preserved grain alone is more effective than butyric acid supplementation or their combination, in improving intestinal health and growth in post-weaned pigs offered low-CP diets.

In this study, no treatment experienced diarrhoea, possibly due to their low CP content, aligning with similar studies [10,11]. Interestingly, OA-preserved grain improved FCR compared to dried grain, with the OA-preserved diet having the best FCR of all treatments. This suggests that OA preservation may counteract the commonly observed decreased FCR observed in low-CP diets [47,48]. The improved FCR of OA-preserved grain diets may be due to improved nutrient digestion. Previous studies have shown that supplementing OA can improve nutrient digestibility in weaned pigs [49,50]. In line with these findings, the current study demonstrated that OA-preserved grain enhanced the CATTD of DM, OM, GE and N, consistent with the work of Maher et al. [17]. Additionally, the increased duodenal villus height observed in the OA-preserved grain diets could further explain the improvements in nutrient digestion and FCR.

While some studies have reported beneficial effects of the individual use of OA-preserved grain and butyric acid supplementation on ADFI [17,51,52], this study found that butyric acid supplementation reduced ADFI during the overall experimental period. Additionally, the interaction between the grain preservation method and butyric acid supplementation resulted in the combination diet having the lowest ADFI from days 15 to 35 of all treatments, without affecting growth. This reduction in feed intake may be due to the high inclusion level of butyric acid used in this study combined with the propionic acid used in the OA-preserved grain, as previous research has shown that higher butyric acid inclusion levels (such as 5%) can modulate appetite-related genes and reduce feed intake [53]. Although reduced feed intake post-weaning is typically associated with impaired mucosal barrier function [54], altered intestinal morphology [55], and compromised growth performance [56], the combination diet showed the highest jejunal VH and greatest relative expression of the glucose transporter *SLC2A1* in the jejunum compared to all other treatments, indicating that intestinal function was not compromised. Moreover, the combination diet exhibited the highest CATTD of DM, GE, N, ash and OM of all treatments, indicating that nutrient digestion was enhanced despite the reduced feed intake. Since butyrate is known to promote satiety [21], the 3% inclusion level may have contributed to the reduced feed intake. Reducing the inclusion level of butyric acid may prevent this reduction in feed intake by reducing the overall acid content while maintaining its beneficial effects on intestinal digestion.

Low-CP diets can reduce intestinal fermentative capacity by reducing SCFA-producing bacteria and their associated metabolites, including butyrate [13]. Given the microbiome’s critical role in promoting intestinal maturation, supporting immune function and improving growth performance in post-weaned pigs [57], this reduction in fermentative capacity could have negative consequences. The metabolites produced by intestinal fermentation, such as SCFAs, are critical for the morphology and function of epithelial cells and can reduce the proliferation of pathogenic bacteria such as *Escherchia coli* [58]. The results of this study support the hypothesis that OA-preserved grain can positively impact intestinal fermentation, promoting a favourable microbiome to support gut health. The OA-preserved diet exhibited the highest abundance of colonic *Rikenellaceae*, *Prevotellaceae*, *Roseburia* and *Alloprevotella* of all treatments, which contribute to gut health through butyrate production and anti-inflammatory effects [59,60,61]. Furthermore, OA-preserved grain increased molar caecal butyrate proportions and total caecal VFA concentrations, while reducing the expression of the pro-inflammatory cytokine *CXCL8* and increasing jejunal *TJP1* expression compared to dried grain. These findings highlight the role of endogenous butyrate in reducing intestinal inflammation [62] and supporting gut development [63]. The effects of OA-preserved grain on jejunal *CXCL8* expression are particularly noteworthy considering the association between low-CP diets and increased intestinal pro-inflammatory cytokine expression [64]. This may be due to the synthetic amino acids used in low-CP being unable to bind with immune cells to stimulate regulatory T cell production [65]. Therefore, OA-preserving low-CP diets could possibly mitigate intestinal inflammation, improve intestinal barrier function and increase total colonic VFA concentrations.

Butyric acid supplementation also demonstrated positive effects on intestinal fermentation, with reductions in colonic Proteobacteria and increases in the abundance of colonic *Spirochaetaceae* and *Treponema* compared to non-butyric acid-supplemented pigs. The overabundance of Proteobacteria is associated with gut dysbiosis [66] and PWD [67], while *Spirochaetaceae* is positively associated with increased pig body weight [68] and *Treponema* are SCFA producers [69] associated with pig feed efficiency [70]. Butyric acid supplementation also increased colonic molar proportions of butyrate and propionate compared to non-butyric acid-supplemented diets. Butyrate suppresses pro-inflammatory cytokines [62], maintains an anaerobic intestinal environment [71] and improves intestinal barrier function [72], while, propionate plays a role in colonic water absorption [73] and pathogens inhibition [74]. These fermentative shifts demonstrate while butyric acid supplementation did not directly affect growth performance; it promoted a beneficial intestinal microbiome that supports gut health and function. Considering the high health status and low bacterial load of the research facility, the potential of butyric acid supplementation to support pig health may be more apparent during an immune challenge situation.

The combination diet had unexpected negative effects on the intestinal fermentative capacity. Specifically, the combination diet exhibited the highest abundance of ileal Proteobacteria and *Pasteruellaceae* of all treatments, both of which are opportunistic pathogens [66,75]. An increased abundance of Proteobacteria is associated with gut dysbiosis [66] and PWD [67], while *Pasteurellaceae* are known to cause serious disease in the respiratory and gastrointestinal tracts [75] and are associated with Crohn’s disease in humans [76]. The high acid content of this combination diet may have disrupted the microbiome, as observed in a similar study where OA supplementation to an OA-preserved grain diet negatively altered the gut microbiome of post-weaned pigs [26]. Moreover, the combination diet had the highest concentrations of colonic BCFAs of all treatments, metabolites which are known to reduce gut barrier function and increase the expression of pro-inflammatory cytokines [77]. These findings suggest that while OA-preserved grain and butyric acid individually have positive effects on intestinal fermentation, their combined use may compromise gut health rather than enhance it.

The result of this study indicates the OA-preserving low CP diets is a more effective strategy than butyric acid supplementation at improving piglet growth and gut health post-weaning. However, given the potential positive effects of butyric acid on intestinal health and function, further research is recommended to identify the optimum inclusion levels of butyric acid supplementation in OA-preserved low-CP diets to maximise its benefits without compromising feed intake and the gut microbiome.

## 5. Conclusions

In conclusion, this study demonstrates that OA preservation is more effective than butyric acid supplementation in supporting the growth and intestinal health of piglets offered low-CP diets post-weaning. The OA-preserved diet had the best FCR, increased abundance of beneficial gut bacteria, the highest caecal butyrate concentrations and the lowest expression of jejunal *CXCL8* of all treatments. Pigs offered OA-preserved grain also had improved nutrient digestibility. Although butyric acid supplementation reduced feed intake, it improved nutrient digestibility, reduced the abundance of colonic Proteobacteria and increased the molar proportions of propionate and butyrate in the colon. However, the combination of OA-preserved grain with butyric acid resulted in the highest abundance of ileal Proteobacteria and *Pasteruellaceae* and the highest colonic BCFA concentrations of all treatments, suggesting potential negative effects on the gut microbiome. Further research is needed to identify the optimum inclusion level of butyric acid in OA-preserved low-CP diets to improve post-weaning growth and intestinal health without compromising feed intake and the gut microbiome.

## Figures and Tables

**Table 1:** The chemical and microbiological analysis of experimental grain after storage (g/kg) unless otherwise stated.

Cereal Crop Type	Wheat		Barley	
Grain Preservation Method	Dried	OA-Preserved	Dried	OA-Preserved
Analysis post storage (g/kg)				
DM	873.5	840.5	873.5	848.5
Ash	16.0	16.0	19.5	19.0
GE (MJ/kg)	15.9	15.3	16.1	15.6
Crude protein	89.0	84.5	103.5	87.5
Crude fibre	25.5	23.5	57.5	52.0
Starch	626.5	608.5	530.0	504.0
Fat	14.0	14.5	15.5	14.0
TMC (cfu/g)	37,000	3800	27,000	2400
Mycotoxin levels (μg/kg) ^a^				
Deoxynivalenol	<75	<75	<75	<75
T-2 toxin	<4.00	<4.00	7.0	<4.00
HT-2 toxin	<4.00	<4.00	30.1	8.7
Zearalenone	<10	<10	<10	<10
Ochratoxin A	3.8	<1.00	1.75	<1.00

Abbreviations: DM, dry matter; GE, gross energy; TMC, total mould count. ^a^ The following mycotoxins were below detectable levels: Aflatoxin B1, B2, G1 and G2.

**Table 2:** Ingredients and chemical composition of dietary treatments (g/kg).

	Dietary Treatments *							
	Stage 1 Diets				Stage 2 Diets			
Grain Preservation Method	Dried	OA-Preserved	Dried	OA-Preserved	Dried	OA-Preserved	Dried	OA-Preserved
Butyric Acid Supplementation	No	No	Yes	Yes	No	No	Yes	Yes
Ingredients (g/kg)								
Wheat	308.0	308.0	305.0	305.0	386.0	386.0	383.0	383.0
Barley	150.0	150.0	150.0	150.0	150.0	150.0	150.0	150.0
Maize	170.0	170.0	170.0	170.0	144.5	144.5	144.5	144.5
Full fat soya	140.0	140.0	140.0	140.0	119.0	119.0	119.0	119.0
Soya bean meal	70.0	70.0	70.0	70.0	59.5	59.5	59.5	59.5
Soya bean concentrate	60.0	60.0	60.0	60.0	51.0	51.0	51.0	51.0
Whey powder	50.0	50.0	50.0	50.0	42.5	42.5	42.5	42.5
Soya oil	30.0	30.0	30.0	30.0	25.5	25.5	25.5	25.5
Salt	2.0	2.0	2.0	2.0	2.0	2.0	2.0	2.0
Mono calcium phosphate	4.2	4.2	4.2	4.2	4.2	4.2	4.2	4.2
Calcium carbonate	4.5	4.5	4.5	4.5	4.5	4.5	4.5	4.5
L-Lysine HCl, 78.8%	4.9	4.9	4.9	4.9	4.9	4.9	4.9	4.9
DL-Methionine	2.5	2.5	2.5	2.5	2.5	2.5	2.5	2.5
L-Threonine	2.7	2.7	2.7	2.7	2.7	2.7	2.7	2.7
Tryptophan	0.7	0.7	0.7	0.7	0.7	0.7	0.7	0.7
Valine	0.5	0.5	0.5	0.5	0.5	0.5	0.5	0.5
Butyric acid	0	0	3.0	3.0	0	0	3.0	3.0

* Dietary treatments: piglets were offered a stage 1 diet (19% CP) from days 0 to 15: (1) dried grain diet; (2) OA-preserved grain diet; (3) dried grain diet + 3 g/kg of encapsulated butyric acid diet; (4) OA-preserved grain + 3 g/kg of encapsulated butyric acid diet. Piglets were then offered a corresponding stage 2 diet (17.5% CP) from days 15 to 35: (1) dried grain diet; (2) OA-preserved grain diet; (3) dried grain + 3 g/kg of encapsulated butyric acid diet; (4) OA-preserved grain + 3 g/kg of encapsulated butyric acid diet.

**Table 3:** The analysed composition of experimental stage 1 and stage 2 diets (g/kg unless otherwise stated).

	Dietary Treatments *							
	Stage 1 Diets				Stage 2 Diets			
Grain Preservation Method	Dried	OA-Preserved	Dried	OA-Preserved	Dried	OA-Preserved	Dried	OA-Preserved
Butyric Acid Supplementation	No	No	Yes	Yes	No	No	Yes	Yes
Ingredients (g/kg)								
DM	896.00	882.50	896.00	884.00	892.50	888.00	891.00	877.00
Ash	32.00	33.50	35.00	35.50	33.50	32.00	30.00	32.00
GE (MJ/kg)	16.65	16.2	17.04	16.75	16.68	16.47	16.98	16.20
Crude fat	58.50	57.00	59.50	58.00	54.00	53.00	52.50	50.00
Crude protein	185.00	182.50	194.00	191.50	172.50	175.00	177.50	177.50
Crude fibre	25.00	23.50	25.50	22.00	25.50	22.00	27.50	25.00
NDF	107.00	98.00	104.50	99.00	106.50	95.00	112.00	102.50
ADF	31.00	28.50	30.00	28.00	30.50	27.00	33.50	31.50
Starch	354.00	350.00	349.00	348.00	383.50	375.50	392.50	380.50
Lysine	15.57	15.55	15.56	15.58	14.24	14.25	14.27	14.24
Threonine	10.71	10.70	10.68	10.71	9.99	9.96	9.96	9.98
Methionine and cysteine	10.03	10.01	10.00	10.03	9.55	9.53	9.52	9.56
Leucine	17.49	17.45	17.47	14.45	14.27	14.30	14.25	14.26
Isoleucine	9.53	9.56	9.55	9.52	8.72	8.76	8.70	8.73
Arginine	12.05	12.06	12.06	12.07	11.10	11.07	11.11	11.07
Histidine	5.18	5.20	5.18	5.22	4.79	4.78	4.77	4.80
Phenylalanine	9.73	9.76	9.72	9.74	9.02	9.04	9.02	9.01
Tyrosine	6.56	6.54	6.58	6.56	6.09	6.07	6.07	6.09
Alanine	9.33	9.36	9.31	9.35	8.53	8.55	8.57	8.56
Aspartic	19.75	19.78	19.77	19.75	17.74	17.77	17.72	17.76
Glutaminc	41.92	41.90	41.89	41.93	40.05	40.01	40.00	40.03
Glycine	7.81	7.78	7.84	7.82	7.33	7.30	7.35	7.31
Serine	9.93	9.96	9.91	9.92	9.23	9.23	9.25	9.24
Proline	14.32	14.34	14.35	19.32	13.75	13.78	13.77	13.80
Tryptophan	2.73	2.74	2.71	2.74	2.62	2.60	2.64	2.62
Valine	10.62	10.66	10.66	10.63	7.83	7.80	7.84	7.81
TMC (cfu/g)	4800	3300	14,000	4000	5600	4000	5200	4700
Mycotoxin levels (mg/kg) ^a^								
Deoxynivalenol	<75	<75	<75	<75	<75	<75	<75	<75
T-2 toxin	<4.00	<4.00	<4.00	<4.00	<4.00	<4.00	<4.00	<4.00
HT-2 toxin	14.10	11.30	14.60	12.10	<10.70	<10.60	9.62	10.70
Zearalenone	30.00	27.00	26.00	22.00	23.00	25.00	25.00	18.00
Ochratoxin	2.39	<1.00	<1.00	<1.00	<1.60	<1.00	1.46	1.43

* Dietary treatments piglets were offered a stage 1 diet (19% CP) from days 0 to 15: (1) dried grain diet; (2) OA-preserved grain diet; (3) dried grain diet + 3 g/kg of encapsulated butyric acid; (4) OA-preserved grain + 3 g/kg of encapsulated butyric acid. Piglets were then offered a corresponding stage 2 diet (17.5% CP) from days 15 to 35: (1) dried grain diet; (2) OA-preserved grain diet; (3) dried grain diet + 3 g/kg of encapsulated butyric acid; (4) OA-preserved grain diet + 3 g/kg of encapsulated butyric acid. Abbreviations: DM, dry matter; GE, gross energy; NDF, neutral detergent fibre; ADF, acid detergent fibre; TMC, total mould count. ^a^ The following mycotoxins were below the listed detectable levels: Aflatoxin B1, B2, G1 and G2 (<1 μg/kg); Fumonisin B1 (<125 μg/kg) and Fumonisin B2 (<50 μg/kg).

**Table 4:** Panel of primer sequences for QPCR analysis.

Target Gene	Gene Name	Accession No.	Forward Primer (5′-3′) Reverse Primer (5′-3′)
Nutrient transporters			
*FABP2*	Fatty Acid Binding Protein 2	NM_001031780.1	F: CAGCCTCGCAGACGGAACTGAA R: GTGTTCTGGGCTGTGCTCCAAGA
*SLC2A1*	Solute Carrier family 2 Member 1	XM_003482115.1	F: TGCTCATCAACCGCAATGA R: GTTCCGCGCAGCTTCTTC
*SLC15A1*	Solute Carrier Family 15 Member 1	NM_214347.1	F: GGATAGCCTGTACCCCAAGCT R: CATCCTCCACGTGCTTCTTGA
Inflammatory markers			
*IL1A*	Interleukin 1A	NM_214029.1	F: CAGCCAACGGGAAGATTCTG R: ATGGCTTCCAGGTCGTCAT
*IL1B*	Interleukin 1B	NM_001005149.1	F: TTGAATTCGAGTCTGCCCTGT R: CCCAGGAAGACGGGCTTT
*IL6*	Interleukin 6	NM_214399.1	F: GACAAAGCCACCACCCCTAA R:CTCGTTCTGTGACTGCAGCTTATC
*CXCL8*	C-X-C motif chemokine ligand 8	NM_213867.1	F: TGCACTTACTCTTGCCAGAACTG R: CAAACTGGCTGTTGCCTTCTT
*IL10*	Interleukin 10	NM_214041.1	F: GCCTTCGGCCCAGTGAA R: AGAGACCCGGTCAGCAACAA
*IL17*	Interleukin 17	NM_001005729.1	F: CCCTGTCACTGCTGCTTCTG R: TCATGATTCCCGCCTTCAC
*IL22*	Interleukin 22	XM_001926156.1	F: GATGAGAGAGCGCTGCTACCTGG R: GAAGGACGCCACCTCCTGCATGT
*TNF*	Tumour Necrosis Factor	NM_214022.1	F: TGGCCCCTTGAGCATCA R: CGGGCTTATCTGAGGTTTGAGA
*FOXP3*	Forkhead box P3	NM_001128438.1	F: GTGGTGCAGTCTCTGGAACAAC R: AGGTGGGCCTGCATAGCA
Tight junctions			
*TJP1*	Tight Junction Protein 1	XM_021098827.1	F: TGAGAGCCAACCATGTCTTGAA R: CTCAGACCCGGCTCTCTGTCT
*CLDN1*	Claudin 1	NM 001244539.1	F: CTGGGAGGTGCCCTACTTTG R: TGGATAGGGCCTTGGTGTTG
Toll like receptors			
*TLR4*	Toll-like Receptor 4	NM_001293317.1	F: TGCATGGAGCTGAATTTCTACAA R: GATAAATCCAGCACCTGCAGTTC
Mucins			
*MUC2*	Mucin 2	AK231524	F: CAACGGCCTCTCCTTCTCTGT R: GCCACACTGGCCCTTTGT
Reference genes			
*H3F3A*	Histone H3.3	NM_213930.1	F: CATGGCTCGTACAAAGCAGA R: ACCAGGCCTGTAACGATGAG
*YWHAZ*	Tyrosine 3-Monooxygenase/tryptophan 5-monooxygenase Activation Protein Zeta	NM_001315726.1	F: GGACATCGGATACCCAAGGA R: AAGTTGGAAGGCCGGTTAATTT
*ACTB*	Actin Beta	XM_001927228.1	F:GGACATCGGATACCCAAGGA R:AAGTTGGAAGGCCGGTTAATTT

**Table 5:** The effect of dietary treatment on pig growth performance and faecal scores (least square means with their standard errors).

Treatment *						*p*-Value		
Grain Preservation Method	Dried	OA-Preserved	Dried	OA-Preserved	SEM	Grain	Butyric	Grain × Butyric
Butyric Acid Supplementation	No	No	Yes	Yes				
**Day 0–15**								
ADFI (g/d)	451	450	419	414	15.86	0.823	**0.030**	0.913
ADG (g/d)	262	366	337	354	20.34	0.603	0.354	0.773
FCR (kg/kg)	1.31	1.25	1.29	1.21	0.057	0.208	0.538	0.921
BW (kg)	12.83	12.90	12.46	12.71	0.305	0.603	0.354	0.773
FS	2.18	2.19	2.17	2.16	0.029	0.981	0.356	0.681
**Day 15–35**								
ADFI (g/d)	862 ^ab^	933 ^b^	879 ^ab^	834 ^a^	26.51	0.611	0.116	**0.028**
ADG (g/d)	551	630	561	553	27.04	0.176	0.210	0.107
FCR	1.65	1.50	1.62	1.54	0.076	0.123	0.970	0.605
BW (kg)	22.74	24.24	22.55	22.67	0.678	0.228	0.189	0.301
**Day 0–35**								
ADFI (g/d)	675	714	670	643	21.77	0.759	**0.047**	0.100
ADG (g/d)	465	515	459	463	20.36	0.179	0.147	0.242
FCR	1.49	1.40	1.49	1.39	**0.047**	0.040	0.992	0.918

Abbreviations: BW, body weight; ADG, average daily gain; ADFI, average daily feed intake; FCR, feed conversion ratio, FS, faecal score; * A total of eight replicates were used per treatment group (replicate = pen, 3 pigs/pen) for the first 8 days, after sample collection on day 8, (replicate = pen, 2 pigs/pen). ^a,b^ Mean values within a row with different superscript letters were significantly different.

**Table 6:** The effect of dietary treatment on the coefficient of apparent total tract digestibility of dry matter (DM), organic matter (OM), ash, nitrogen (N) and gross energy (GE) on day 30 post-weaning (least square means with their SEM).

	Treatment *				SEM	*p*-Value		
Grain Preservation Method	Dried	OA-Preserved	Dried	OA-Preserved		Grain	Butyric	Grain × Butyric
Butyric Acid Supplementation	No	No	Yes	Yes				
DM	0.845	0.852	0.847	0.865	0.0040	**0.005**	0.063	0.181
OM	86.30	86.88	86.47	88.00	0.383	**0.008**	0.095	0.216
Ash	57.70 ^ab^	59.87 ^b^	56.72 ^a^	63.71 ^c^	1.091	<0.010	0.186	**0.031**
N	78.41	80.89	79.96	82.23	0.710	**0.002**	**0.045**	0.878
GE	83.68	84.57	84.26	85.77	0.422	**0.007**	**0.039**	0.455

Abbreviations: DM, dry matter; OM, organic matter; N, nitrogen; GE, gross energy. * A total of eight replicates were used per treatment group; SEM, standard error of the mean. ^a,b,c^ Mean values within a row with different superscript letters were significantly different.

**Table 7:** The effect of dietary treatment on small intestinal morphology (least square means with their standard errors).

	Treatment *				SEM	*p*-Value		
Grain Preservation Method	Dried	OA-Preserved	Dried	OA-Preserved		Grain	Butyric	Grain × Butyric
Butyric Acid Supplementation	No	No	Yes	Yes				
Duodenum								
VH μm	271.79	303.53	244.60	308.73	20.257	**0.023**	0.587	0.440
CD μm	124.66	134.99	88.94	114.58	9.435	0.063	**0.006**	0.433
VH:CD	2.29	2.28	2.93	2.81	0.267	0.802	**0.034**	0.844
Jejunum								
VH μm	307.42 ^a^	303.48 ^ab^	250.46 ^a^	366.93 ^b^	24.592	0.028	0.895	**0.024**
CD μm	135.23	106.17	101.81	121.26	13.119	0.712	0.486	0.081
VH:CD	2.46	2.88	2.61	3.11	0.261	0.082	0.472	0.892
Ileum								
VH μm	294.51	297.82	285.33	299.50	17.489	0.615	0.830	0.763
CD μm	102.70	93.09	98.73	103.09	7.536	0.725	0.689	0.372
VH:CD	2.90	3.32	2.93	3.05	0.242	0.266	0.622	0.549

Abbreviations: VH, villus height; CD, crypt depth; VH:CD villus height to crypt depth ratio. * A total of eight replicates were used per treatment group. ^a,b^ Mean values within a row with different superscript letters were significantly different.

**Table 8:** The effect of dietary treatment on the relative expression of genes involved in nutrient transportation and inflammation that were differentially expressed in the small intestine (least square means with their standard errors.

	Treatment *				SEM	*p*-Value		
Grain Preservation Method	Dried	OA-Preserved	Dried	OA-Preserved		Grain	Butyric	Grain × Butyric
Butyric Acid Supplementation	No	No	Yes	Yes				
Duodenum								
*IL1A*	1.58	0.93	0.96	1.50	0.272	0.829	0.932	**0.029**
*MUC2*	1.04 ^ab^	0.88 ^a^	0.94 ^a^	1.26 ^b^	0.125	0.517	0.256	**0.050**
Jejunum								
*SLC2A1*	1.00 ^a^	0.99 ^a^	0.94 ^a^	1.36 ^b^	0.097	0.040	0.125	**0.036**
*CXCL8*	1.13	0.87	1.38	0.91	0.180	**0.042**	0.418	0.556
*TJP1*	0.93	1.03	0.94	1.04	0.040	**0.025**	0.866	0.939
Ileum								
*TLR4*	1.22 ^a^	0.90 ^ab^	0.78 ^b^	1.24 ^a^	0.131	0.580	0.688	**0.006**

*IL1A*, interleukin 1A; *MUC2*, mucin 2; *SLC2A1*, glucose transporter member 1; *CXCL8*, C-X-C motif chemokine ligand 8; *TJP1*, tight junction protein 1; *TLR4*, toll like receptor 4. * A total of eight replicates were used per treatment. ^a,b^ Mean values within a row with different superscript letters were significantly different.

**Table 9:** The effect of dietary treatment on the relative abundance of selected bacterial phyla in the ileal and colonic digesta (mean % relative abundance with their standard errors).

Phylum	Treatments *					*p*-Value		
Grain Preservation Method	Dried	OA-Preserved	Dried	OA-Preserved	SEM	Grain	Butyric	Grain × Butyric
Butyric Acid Supplementation	No	No	Yes	Yes				
Ileum								
Firmicutes	80.46 ^a^	91.20 ^ab^	98.77 ^b^	82.67 ^ab^	4.271	0.582	0.270	**0.005**
Proteobacteria	19.54 ^a^	1.61 ^b^	0.87 ^b^	12.35 ^c^	1.977	0.783	0.082	**<0.001**
Colon								
Firmicutes	76.98	70.84	74.17	77.90	3.121	0.680	0.485	0.117
Bacteroidetes	8.53 ^a^	17.63 ^b^	11.34 ^a^	10.72 ^a^	1.484	0.004	0.323	**0.001**
Actinobacteria	5.50	3.65	5.28	2.32	0.829	**0.002**	0.188	0.271
Tenericutes	0.48	1.71	0.47	2.34	0.541	**0.001**	0.716	0.684
Proteobacteria	2.97	1.35	0.07	0.51	0.610	0.409	**0.004**	0.070
Spirochaetes	1.59	0.49	5.33	1.36	0.816	**0.001**	**0.002**	0.783

* A total of eight replicates were used per treatment group. ^a,b,c^ Mean values within a row with unlike superscripts letters were significantly different (*p* < 0.05).

**Table 10:** The effect of dietary treatments on the relative abundance of selected bacterial families in the ileal and colonic digesta (mean % relative abundance with their standard errors).

Family	Treatments *					*p*-Value		
Grain Preservation Method	Dried	OA-Preserved	Dried	OA-Preserved	SEM	Grain	Butyric	Grain × Butyric
Butyric Acid Supplementation	No	No	Yes	Yes				
Ileum								
*Lactobacillaceae*	79.18	77.07	81.49	73.90	4.450	0.242	0.899	0.499
*Clostridiaceae*	4.77 ^a^	6.15 ^a^	12.29 ^b^	1.77 ^c^	1.431	0.002	0.509	**<0.001**
*Streptococcaceae*	0.39 ^a^	8.43 ^b^	4.99 ^b^	6.72 ^b^	1.298	<0.001	0.008	**0.002**
*Pasteurellaceae*	0.39 ^a^	1.16 ^a^	0.45 ^a^	9.32 ^b^	1.365	**0.001**	**0.047**	0.079
Colon								
*Lactobacillaceae*	8.46	6.94	8.52	9.72	1.100	0.790	0.171	0.189
*Lachnospiraceae*	12.28	10.76	13.65	11.49	1.320	0.156	0.417	0.847
*Erysipelotrichaceae*	0.35	0.66	0.54	0.57	0.287	0.488	0.773	0.564
*Eubacteriaceae*	3.07	3.44	3.67	3.76	0.701	0.726	0.496	0.828
*Ruminococcaceae*	37.23	34.12	33.64	38.81	2.203	0.641	0.816	0.061
*Clostridiaceae*	2.59	3.59	4.46	2.88	0.746	0.786	0.431	0.067
*Propionibacteriaceae*	5.27	3.36	5.40	1.69	0.822	**<0.001**	0.106	0.084
*Streptococcaceae*	0.14	0.67	0.92	0.07	0.363	0.562	0.831	0.125
*Oscillospiraceae*	2.06	2.15	2.87	2.01	0.599	0.520	0.598	0.417
*Sphingobacteriaceae*	0.09	0.10	0.25	0.09	0.176	0.692	0.687	0.606
*Spiroplasmataceae*	0.45	1.20	0.23	0.99	0.414	**0.026**	0.414	0.656
*Rikenellaceae*	1.22 ^a^	4.51 ^b^	2.58 ^ab^	1.23 ^a^	0.751	0.297	0.307	**<0.001**
*Hungateiclostridiaceae*	2.21	3.22	1.26	2.41	0.634	**0.048**	0.097	0.580
*Muribaculaceae*	0.32	0.45	0.26	0.26	0.236	0.781	0.573	0.801
*Acidaminococcaceae*	0.59	0.65	0.44	0.83	0.322	0.449	0.961	0.571
*Veillonellaceae*	0.19	0.78	0.21	0.18	0.313	0.396	0.354	0.315
*Prevotellaceae*	7.11 ^a^	13.40 ^b^	8.77 ^a^	9.33 ^ab^	1.294	0.006	0.520	**0.021**
*Christensenellaceae*	2.16	1.09	1.37	1.60	0.556	0.381	0.893	0.167
*Spirochaetaceae*	1.66	0.50	3.86	1.39	0.743	**0.003**	**0.010**	0.797
*Coriobacteriacea*	0.45	0.16	0.13	0.29	0.238	0.870	0.682	0.254
*Eubacteriaceae*	3.07	3.44	3.67	3.76	0.701	0.726	0.496	0.828
*Anaeroplasmataceae*	0.05	0.11	0.27	0.02	0.184	0.618	0.933	0.321

* A total of eight replicates were used per treatment group. ^a,b,c^ Mean values within a row with unlike superscript letters were significantly different (*p* < 0.05).

**Table 11:** The effect of dietary treatment on the relative abundance of selected bacterial genera in the ileal and colonic digesta (mean % relative abundance with their standard errors).

Genus	Treatments *					*p*-Value		
Grain Preservation Method	Dried	OA-Preserved	Dried	OA-Preserved	SEM	Grain	Butyric	Grain × Butyric
Butyric Acid Supplementation	No	No	Yes	Yes				
Ileum								
*Lactobacillus*	66.01 ^a^	77.75 ^ab^	82.21 ^b^	74.88 ^ab^	3.943	0.499	0.091	**0.022**
*Clostridium*	4.82 ^a^	6.24 ^a^	12.37 ^b^	2.05 ^a^	1.436	0.002	0.698	**<0.001**
*Streptococcus*	0.39 ^a^	8.51 ^b^	5.01 ^b^	7.79 ^b^	1.305	<0.001	0.005	**0.003**
Colon								
*Lactobacillus*	8.75	7.01	8.67	9.71	1.102	0.658	0.205	0.180
*Collinsella*	0.46	0.16	0.13	0.29	0.240	0.869	0.672	0.250
*Catenibacterium*	0.08	0.13	0.11	0.03	0.129	0.802	0.692	0.551
*Gemmiger*	7.09 ^ab^	5.27 ^a^	5.54 ^ab^	9.27 ^b^	1.077	0.440	0.264	**0.007**
*Ruminococcus*	2.14	1.21	2.23	0.65	0.564	**0.010**	0.380	0.319
*Faecalibacterium*	24.44	24.63	21.95	26.12	1.807	0.218	0.737	0.257
*Butyricicoccus*	1.03	0.81	1.36	1.40	0.418	0.752	0.227	0.694
*Holdemanella*	0.20	0.25	0.25	0.19	0.187	0.974	0.967	0.757
*Clostridium*	1.65	2.69	3.12	1.74	0.625	0.845	0.690	0.139
*Streptococcus*	0.14	0.66	0.92	0.07	0.362	0.559	0.827	0.225
*Oscillibacter*	2.13	2.16	2.91	2.81	0.603	0.958	0.215	0.920
*Spiroplasma*	0.46	1.21	0.23	1.00	0.416	**0.025**	0.405	0.645
*Propionibacterium*	5.58	3.36	4.37	1.68	0.835	**0.001**	**0.023**	0.279
*Anaerocella*	1.22 ^a^	3.19 ^b^	2.58 ^ab^	1.23 ^a^	0.631	0.689	0.705	**0.004**
*Pseudobutyrivibrio*	0.46	0.71	0.38	0.44	0.297	0.580	0.535	0.776
*Eubacterium*	3.16	3.45	3.69	3.76	0.702	0.788	0.535	0.858
*Dorea*	2.09	1.10	3.53	1.46	0.665	**0.010**	0.156	0.657
*Anaerobacterium*	1.72	2.60	1.18	2.09	0.571	0.076	0.271	0.765
*Prevotella*	6.30	7.70	5.73	6.15	0.981	0.341	0.265	0.645
*Phascolarctobacterium*	0.58	0.65	0.45	0.46	0.305	0.906	0.558	0.922
*Roseburia*	1.72	4.38	2.95	3.61	0.740	**0.012**	0.419	0.094
*Fournierella*	0.58	1.48	1.10	0.69	0.430	0.546	0.875	0.079
*Megasphaera*	0.13	0.49	0.58	0.18	0.269	0.930	0.745	0.101
*Agathobacter*	1.11	1.34	0.25	1.56	0.441	**0.031**	0.140	0.075
*Alloprevotella*	0.76 ^a^	3.67 ^b^	2.63 ^b^	2.94 ^b^	0.724	0.004	0.071	**0.012**
*Blautia*	3.13	1.09	1.68	0.25	0.626	**0.002**	**0.021**	0.328
*Prevotellamassilia*	0.47	0.30	0.08	0.27	0.241	0.652	0.273	0.322
*Kineothrix*	1.06	0.30	0.20	0.67	0.390	0.959	0.467	0.143
*Christensenella*	2.14	1.09	1.37	1.60	0.554	0.386	0.910	0.173
*Pseudoflavonifractor*	1.77 ^a^	0.68 ^ab^	0.43 ^b^	1.11 ^ab^	0.503	0.982	0.268	**0.029**
*Solitalea*	0.09	0.11	0.25	0.09	0.176	0.703	0.699	0.593
*Paramuribaculum*	0.07	0.06	0.14	0.05	0.134	0.662	0.849	0.740
*Methanobrevibacter*	1.04	0.63	0.59	0.83	0.386	0.839	0.729	0.326
*Asteroleplasma*	0.05	0.11	0.27	0.02	0.185	0.618	0.930	0.318
*Treponema*	1.48	0.46	3.72	0.99	0.729	**0.002**	**0.026**	0.845

* A total of eight replicates were used per treatment group. ^a,b^ Mean values within a row with unlike superscript letters were significantly different (*p* < 0.05).

**Table 12:** The effect of dietary treatment on the molar proportions and total concentrations of VFA in the caecum and colon (least square means with their standard errors).

	Treatment *					*p*-Value		
Grain Preservation Method	Dried	OA-Preserved	Dried	OA-Preserved	SEM	Grain	Butyric	Grain × Butyric
Butyric Acid Supplementation	No	No	Yes	Yes				
Caecum (mol/g)								
Acetate	0.505	0.450	0.429	0.429	0.0175	0.112	**0.007**	0.1101
Propionate	0.280	0.265	0.328	0.321	0.0138	0.399	**<0.001**	0.749
Butyrate	0.161	0.202	0.184	0.200	0.0126	**0.024**	0.397	0.308
Valerate	0.032	0.039	0.030	0.027	0.0037	0.593	0.056	0.177
Isobutyrate	0.010 ^a^	0.021 ^b^	0.016 ^ab^	0.012 ^a^	0.0025	0.198	0.449	**0.007**
Isovalerate	0.011 ^a^	0.024 ^b^	0.013 ^a^	0.011 ^a^	0.0024	0.078	0.059	**0.008**
BCFA	0.046	0.059	0.062	0.047	0.0066	0.923	0.735	0.131
Total	142.05	153.38	141.05	181.44	10.255	**0.013**	0.177	0.148
Colon (mol/g)								
Acetate	0.512	0.499	0.434	0.421	0.0124	0.330	**<0.001**	0.988
Propionate	0.281	0.298	0.334	0.326	0.0108	0.667	**<0.001**	0.249
Butyrate	0.145	0.146	0.184	0.192	0.0098	0.644	**<0.001**	0.746
Valerate	0.032	0.032	0.027	0.028	0.0040	0.817	0.293	0.914
Isobutyrate	0.015 ^ab^	0.012 ^b^	0.011 ^b^	0.018 ^a^	0.0021	0.341	0.550	**0.033**
Isovalerate	0.017	0.012	0.010	0.014	0.0024	0.958	0.378	0.076
BCFA	0.063 ^a^	0.052 ^a^	0.058 ^a^	0.083 ^b^	0.0065	0.263	0.057	**0.010**
Total	162.47	196.79	194.71	185.50	10.927	0.261	0.346	0.050

Abbreviations: VFA, volatile fatty acids; BCFA, branched-chain fatty acids. * A total of eight replicates were used per treatment group. ^a,b^ Mean values within a row with unlike superscript letters were significantly different (*p* < 0.05).

## Data Availability

The data presented in this study are available on request from the corresponding author.
